# Next-generation sequencing-based microRNA profiling of mice testis subjected to transient heat stress

**DOI:** 10.18632/oncotarget.22900

**Published:** 2017-12-04

**Authors:** Meng Rao, Zhengyan Zeng, Li Tang, Guiping Cheng, Wei Xia, Changhong Zhu

**Affiliations:** ^1^ Department of Reproduction and Genetics, The First Affiliated Hospital of Kunming Medical University, Kunming, China; ^2^ Department of Neurology, The First Affiliated Hospital of Kunming Medical University, Kunming, China; ^3^ Family Planning Research Institute, Tongji Medical College, Huazhong University of Science and Technology, Wuhan, China

**Keywords:** heat stress, spermatogenesis, microRNA, next-generation sequencing, apoptosis

## Abstract

This study aimed to investigate the role of microRNA (miRNA) in heat stress-induced spermatogenic impairment. Testes from 15 adult ICR mice subjected to testicular hyperthermia at 43°C for 30 min and from 15 control mice were collected and pooled into 3 samples. Isolated RNA from these samples was subjected to small RNA high-throughput sequencing, and differentially expressed miRNAs were identified and validated using RT-PCR. The identified miRNAs were further subjected to Gene Ontology and KEGG analyses, which revealed significant enrichment for pathways potentially involved in heat stress-induced spermatogenic impairment. Additionally, a correlation analysis of the relative levels of validated miRNAs with germ cell apoptosis was performed. Of the 11 miRNAs identified as differentially expressed, 8 were validated as consistent with sequencing data. Further analyses suggested that the target genes of those miRNAs were involved in various pathways (e.g., ribosomal, HIF-1, MAPK) that may be critical to heat stress-induced testicular damage. Some identified miRNAs, including miR-449a-3p, miR-92a-1-5p, miR-423-3p, and miR-128-3p, correlated closely with germ cell apoptosis. The study results reveal a detailed miRNA profile of heat stress-induced testicular damage and highlight new and potentially important candidate targets in the process of male infertility.

## INTRODUCTION

In most mammals, including humans, normal spermatogenesis requires suitable environmental conditions, defined as a temperature approximately 2–4°C lower than that of the body core [1]. Scrotal heat stress is a common biological factor that may cause spermatogenic impairment and infertility. Although cryptorchidism and varicocele are the main endogenous factors that promote testicular hyperthermia and subsequently affect normal spermatogenesis [2, 3], workers such as electric welders and drivers, who are exposed to high occupational environmental temperatures, may experience the deleterious effect of spermatogenesis consequent to scrotal heat stress [4, 5]. Additionally, men who regularly used hot springs and saunas were found to have lower fertility levels [6, 7].

According to studies of mice, rats, monkeys, and humans, heat stress-induced spermatogenic impairment mainly results from germ cell apoptosis via the mitochondrial-dependent pathway [8–11]. However, cell apoptosis remains a very complicated process regulated by several known and unknown factors, and the underlying molecular mechanism remains largely unknown. Notably, microRNAs (miRNAs), a class of small non-coding RNAs, are involved in a variety of biological processes, including cell proliferation, apoptosis and tumorigenesis, via the post-transcriptional regulation of gene expression [12–14]. Studies have also demonstrated the importance of miRNAs in the regulation of spermatogenesis, particularly germ cell meiosis, apoptosis, and Sertoli cell function [15–17].

Most studies regarding heat stress and spermatogenesis have focused on protein levels, but none have evaluated the regulatory effects of miRNAs on the heat-induced impairment of spermatogenesis. In the present study, therefore, we performed the first high-throughput sequencing analysis of small RNAs in mouse testes exposed to transient heat stress. Here, we identified differentially expressed miRNAs and applied a bioinformatic analysis to determine the critical target and pathways involved in the heat stress-induced impairment of spermatogenesis. Previous study has revealed that germ cell degeneration, apoptosis, and altered testicular histology were observed as early as 6 hours after heat exposure [18]. Therefore, in this study, we evaluated heat-induced changes in miRNA expression at 6 hours after heat treatment.

## RESULTS

### Germ cell apoptosis

**Figure 1:**
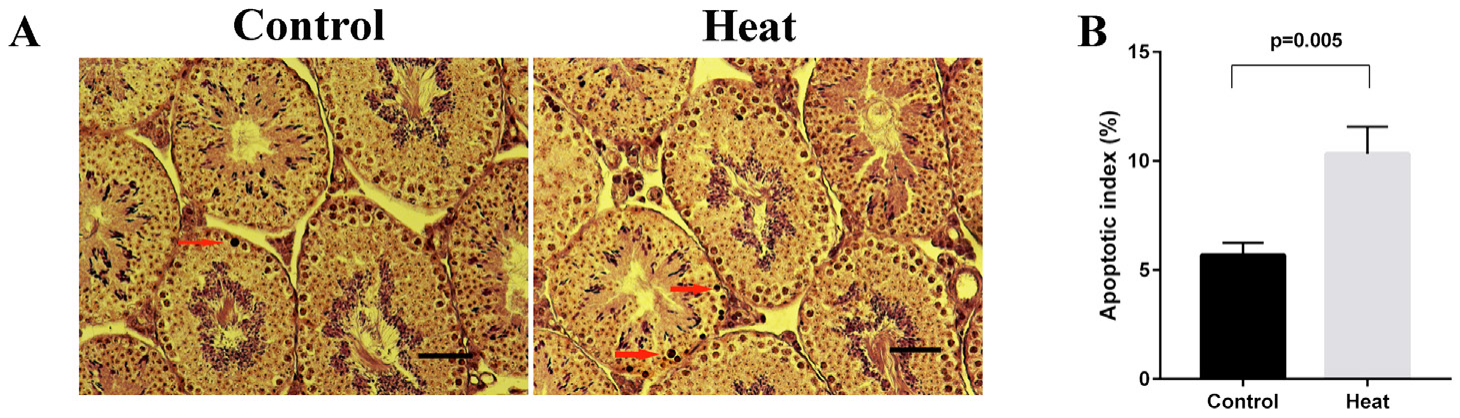
Germ cell apoptosis in control and heat treatment group **(A)** Representative pictures of TUNEL assay on mice testis. Apoptotic cells (arrows) were stained dark brown in nucleus. **(B)** Apoptotic index (AI) was significantly higher than that in control group (*p* = 0.005). Bar=50 μm.

After evaluating germ cell apoptosis, we calculated the AIs. As shown in Figure 1, the testes from the heat treatment group contained more apoptotic germ cells (brown nuclei), compared to those from the control group. Accordingly, the AI was significantly higher in the heat treatment group (10.3%) than in the control group (5.7%) (*p* = 0.005). Notably, severe histological damage was not observed at 6 hours after heat treatment. Accordingly, we selected this time point for further miRNA sequencing to identify differentially expressed miRNAs.

### Overview of next-generation deep sequencing of miRNA data

**Figure 2:**
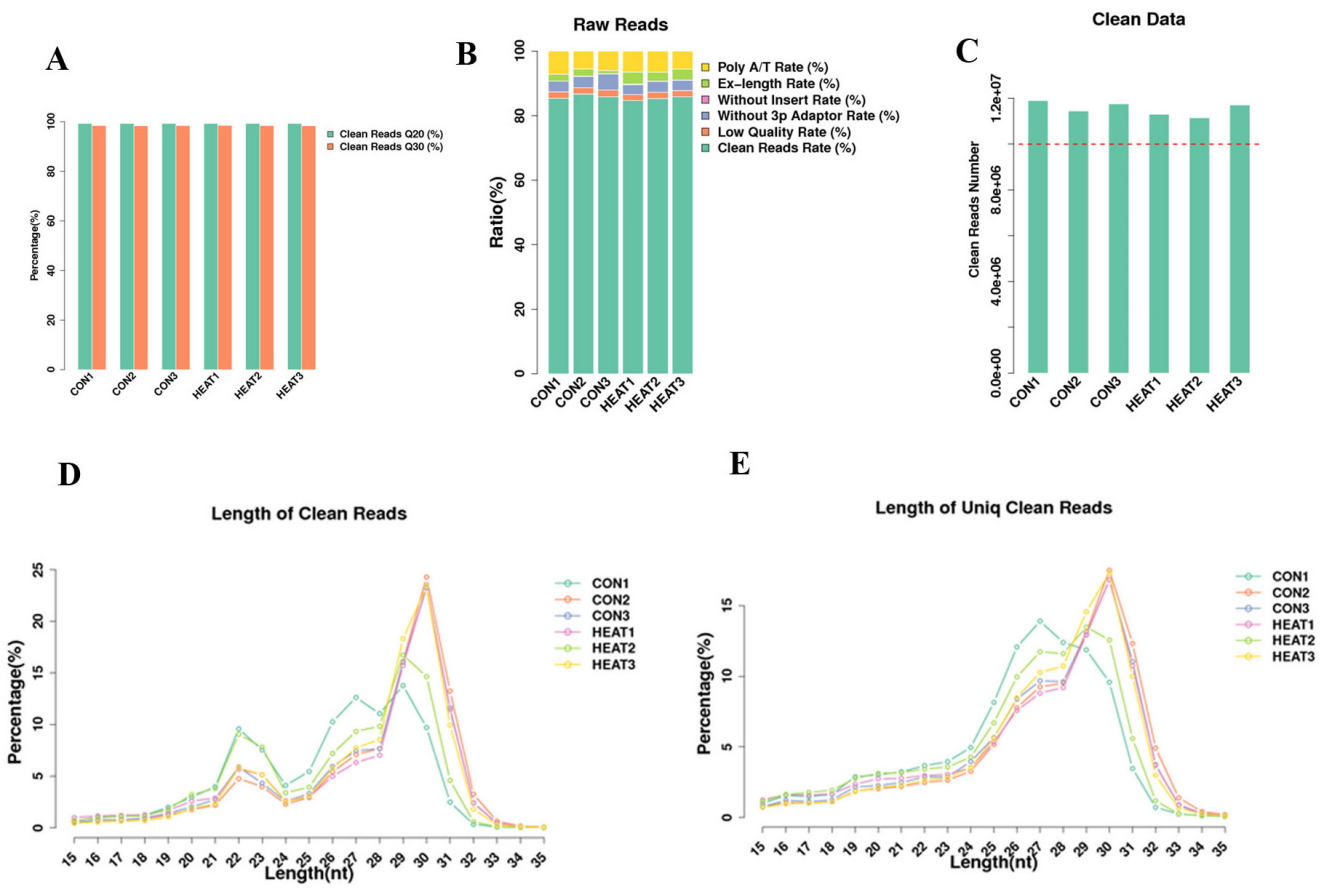
Basic information of raw and clean reads of sequencing data **(A)** The Q20 and Q30 statistics of the clean reads in all samples. **(B)** The composition of those reads and **(C)** the number of obtained clean reads. **(D-E)** The length distribution of the small RNAs. CON, control.

In this study, we subjected 3 pooled controls and 3 pooled treatment samples to high-through output sequencing. After library construction and sequencing, we obtained approximately 13.1–13.9 million raw reads in different samples. Next, the raw data were filtrated to get clean data. The Q20 and Q30 statistics of the clean reads in all samples exceeded 99.2% and 98.4%, respectively (Figure 2A). The composition of those reads and the number of obtained clean reads were shown in Figure 2B and 2C, respectively.

**Figure 3:**
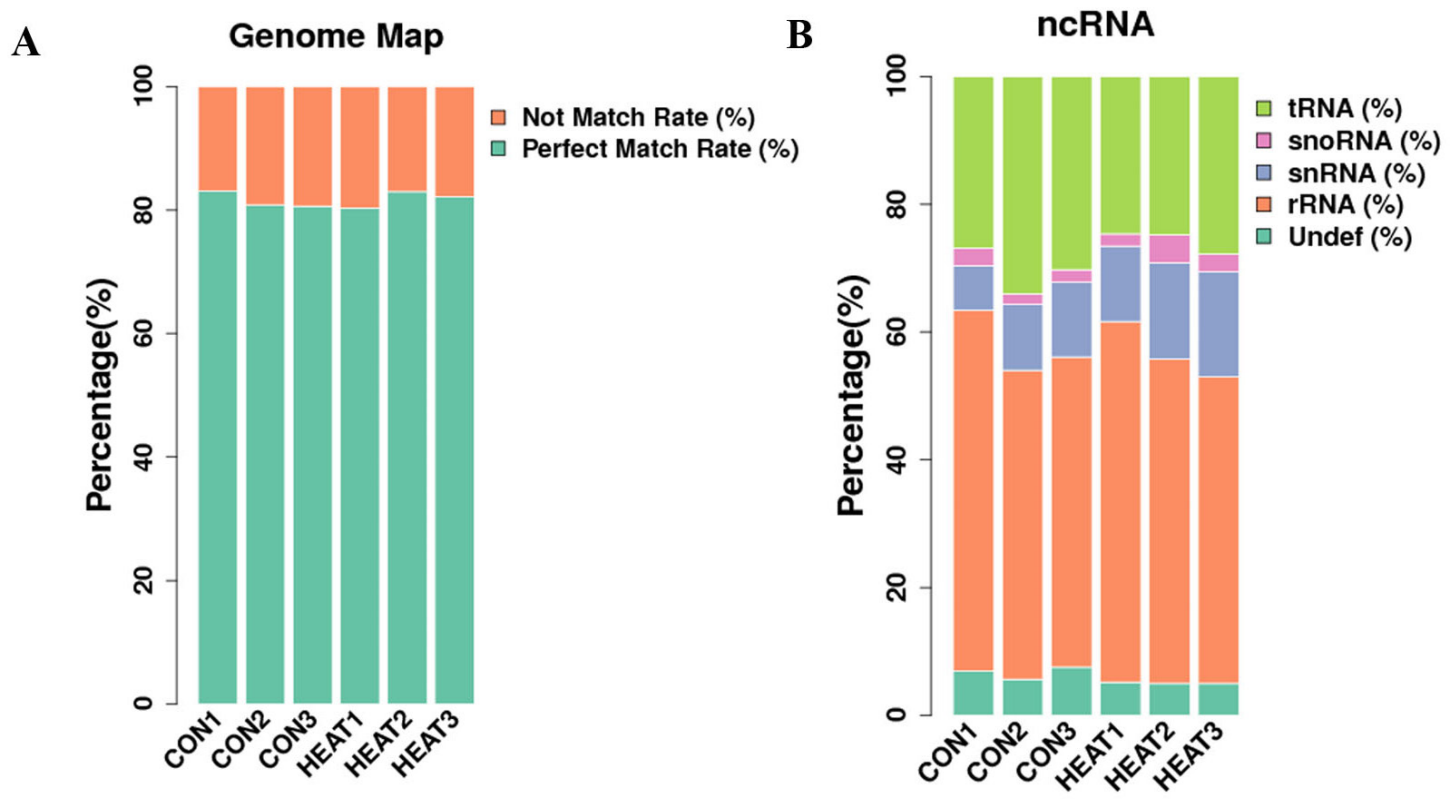
Information of reads mapping to the reference genome sequence **(A)**, and categories of all identified small RNAs **(B)**. ncRNA, non-coding RNA; CON, control.

Next, we analyzed the length distribution of the small RNAs. As shown in Figure 2D–2E, we observed 2 peaks in the length distribution of clean reads, at 22 nucleotides (nt) (miRNA) and 30 nt (Piwi-interacting RNA; piRNA). Thereafter, we mapped reads with lengths of 17–35 nt to the genome using Bowtie software (Version 1.1.2). After mapping 80% of the reads perfectly mapped to the reference genome sequence (Figure 3A), these small RNAs were then classified into several different categories according to their annotations. The sequences of tRNA, snoRNA, snRNA, rRNA, and other kinds of non-coding RNAs (Undef) were identified using a basic local alignment search tool against known non-coding RNAs deposited in the Rfam database and NCBI databases [19, 20], separated from miRNAs, and discarded (Figure 3B).

**Figure 4:**
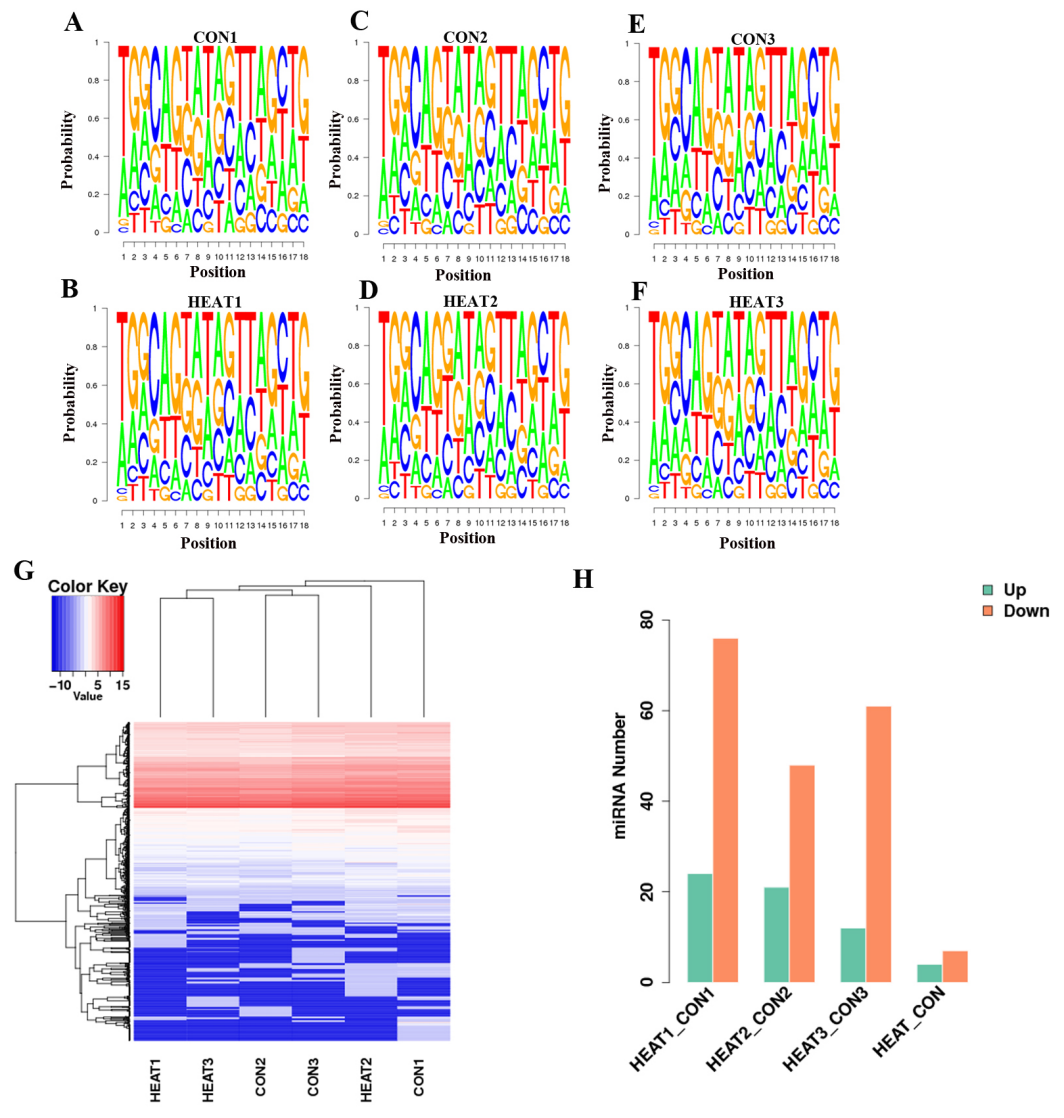
Deep analysis of sequencing of miRNA data The percentages of the four nucleotides at each position of the identified miRNAs is presented in **(A–F)**. **(G)** presents a hierarchical clustering of all differentially regulated miRNAs in the six independent pooled samples. Number of differentially expressed miRNAs are presented in **(H)**. CON, control.

Finally, 536, 475, and 494 known miRNAs were identified in the control 1, 2 and 3 samples, respectively, and 495, 519, and 483 were identified in the heat treatment 1, 2 and 3 samples, respectively. An analysis of the percentages of the four nucleotides at each position of the identified miRNAs is presented in Figure 4A–4F). The base bias data were helpful for further analyses of the miRNA structural characteristics.

### Differentially expressed miRNAs

The hierarchical clustering heatmap shown in Figure 4G depicts presents a hierarchical clustering of all differentially regulated miRNAs in the six independent pooled samples according to the criteria of a fold change >2 and p value <0.05. The global miRNA profile analysis revealed that100, 69, and 73 miRNAs were differentially expressed in the respective pairwise comparisons: control 1 vs. heat 1, control 2 vs. heat 2, and control 3 vs. heat 3. Eleven miRNAs were differentially expressed in the inter-group comparison (CONTROL vs. HEAT); of these, 4 miRNAs were upregulated and 7 were downregulated (Figure 4H). Detailed information about these 11 miRNAs are presented in Table 1.

**Table 1:** Differentially expressed miRNAs

MiRNA	Fold change	p Value	Up/Down
miR-449a-3p	0.231	0.001	down
miR-298-5p	0.369	0.000	down
miR-92a-1-5p	0.372	0.001	down
miR-423-5p	0.414	0.007	down
miR-423-3p	0.425	0.000	down
miR-128-3p	0.432	0.047	down
miR-340-3p	0.475	0.040	down
miR-98-5p	2.025	0.004	up
miR-3968	2.550	0.003	up
miR-21a-3p	2.770	0.015	up
miR-201-3p	3.038	0.022	up

### GO enrichment and KEGG pathway analyses

**Figure 5:**
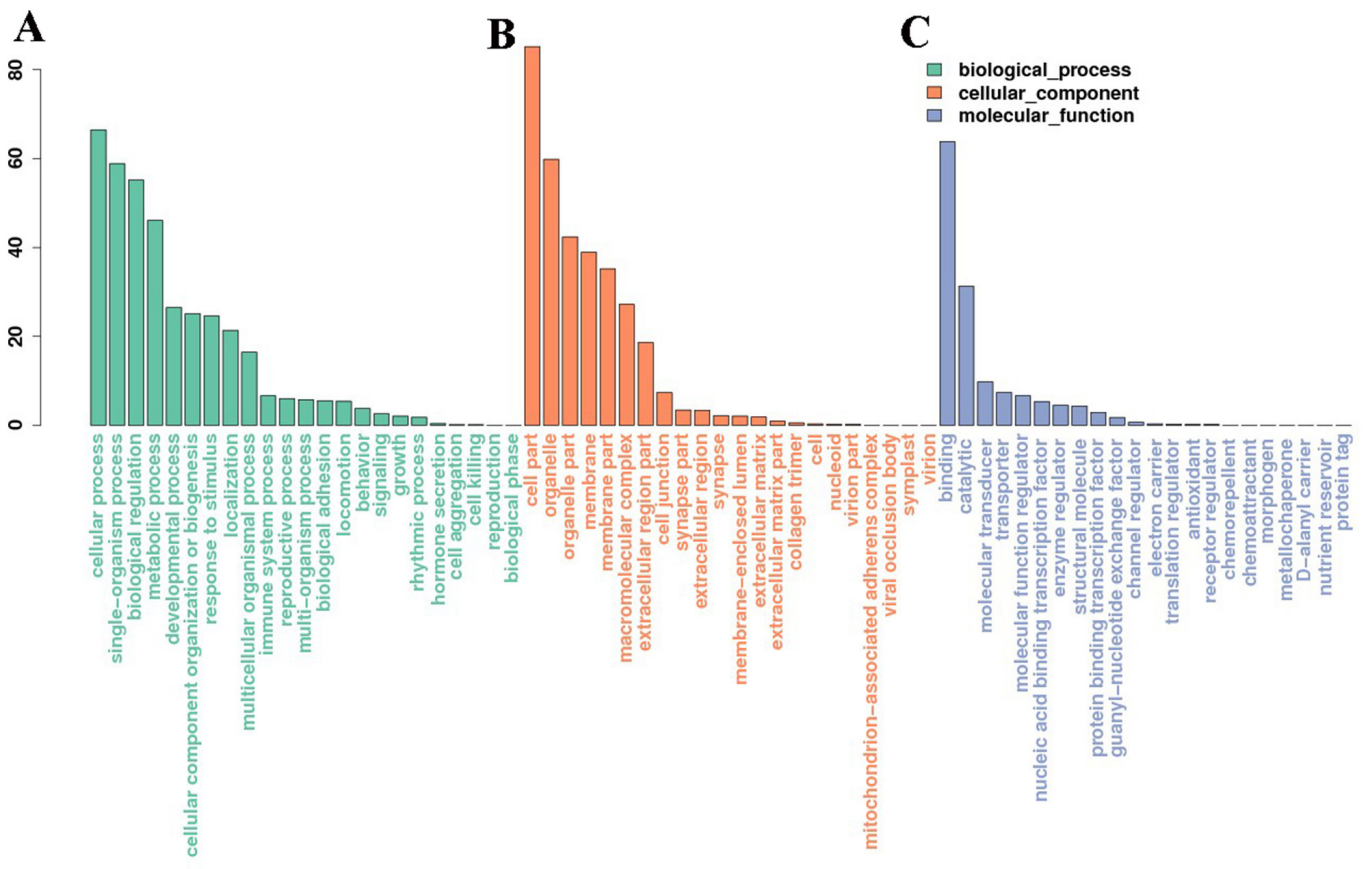
Gene ontology (GO) analysis of the miRNA target genes **(A, B and C)** represent the enriched biological processes, cell components, and molecular functions, respectively (*p* < 0.05).

We used a well-established miRNA-target database (miRanda, 3.3a) to predict the target genes of the identified miRNAs and investigated the possible functions using gene function annotation methods. A GO analysis was conducted to understand the functional roles of the target genes, and an enrichment analysis based on the hypergeometric test was used to test whether a GO term was statistically enriched for a given set of genes. A total of 5293 genes were predicted and enrolled in the GO analysis, and the enriched biological processes, cell components, and molecular functions (*p* < 0.05) are shown in Figure 5. A KEGG pathway analysis was conducted to enrich the potential pathways of the predicted genes. Several pathways, including ribosomal pathways, the hypoxia-induced factor 1 (HIF-1) signaling pathway, mitogen-activated protein kinase (MAPK) and oxidative phosphorylation etc., were significantly enriched in the testes after scrotal hyperthermia (Supplementary Figure 1).

### Validation of differentially expressed miRNAs using qRT-PCR

**Figure 6:**
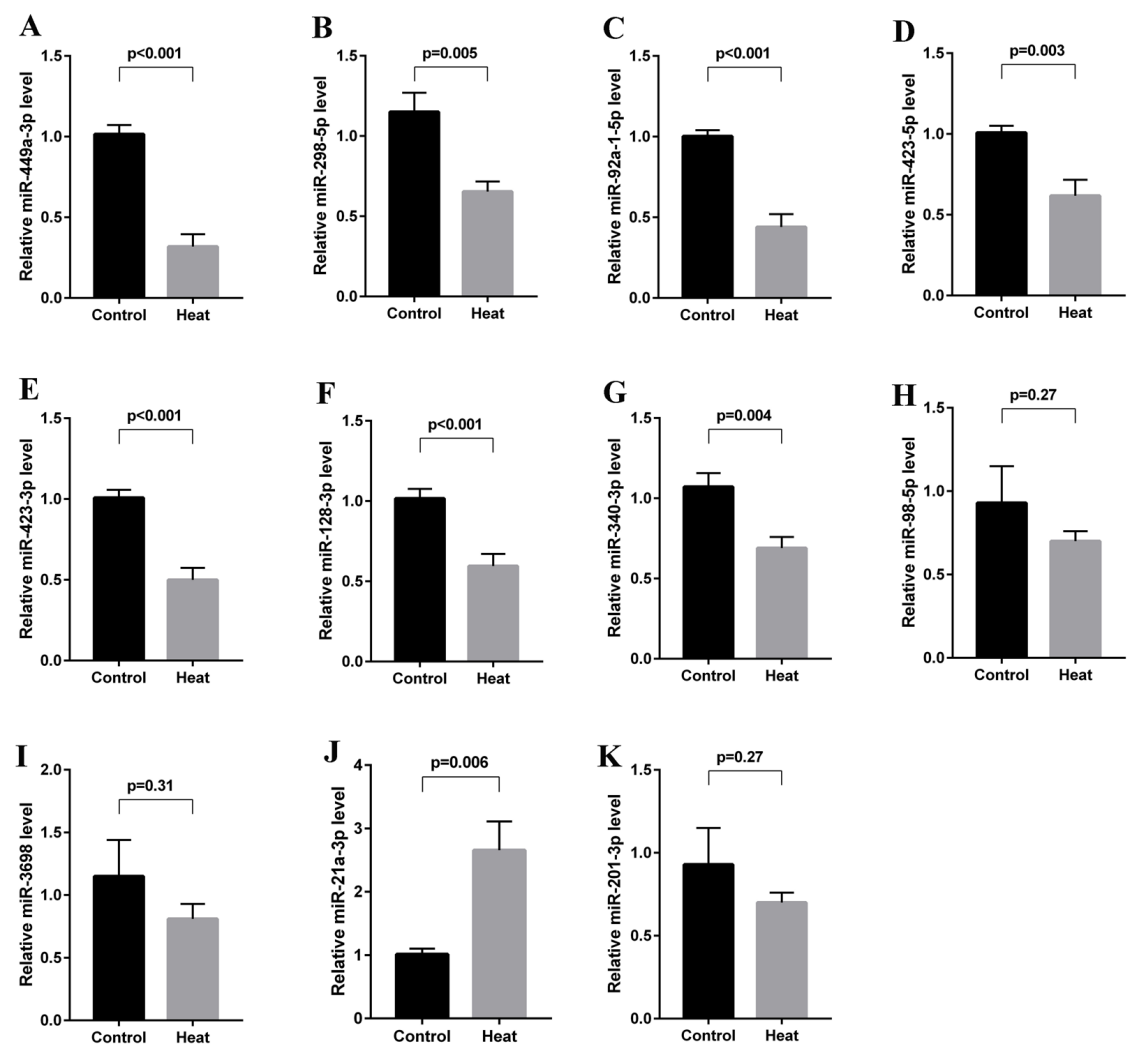
Validation of differentially expressed miRNAs using qRT-PCR All 11 differentially expressed miRNAs were validated in 10 animals each from the control and heat treatment groups. MiR-449a-3p, miR-298-5p, miR-92a-1-5p, miR-423-5p, miR-423-3p, miR-128-3p and miR-340-3p were found to be significantly downregulated (**A-G**, all *p* < 0.05), and miR-21a-3p was found to be significantly upregulated at 6 hours after heat treatment (**J**, *p* = 0.006). MiR-98-5p, miR-3968 and miR-201-3p expression levels were not found to be significantly altered **(H, I, K)**.

All 11 differentially expressed miRNAs were validated in 10 animals each from the control and heat treatment groups using qRT-PCR. Seven of these miRNAs were found to be significantly downregulated (miR-449a-3p, miR-298-5p, miR-92a-1-5p, miR-423-5p, miR-423-3p, miR-128-3p, miR-340-3p), and 1 was found to be significantly upregulated (miR-21a-3p) at 6 hours after transient scrotal heat treatment. The results were consistent with our sequencing data. However, as shown in Figure 6, the expression levels of 3 of the 11 previously identified miRNAs (miR-98-5p, miR-3968 and miR-201-3p) were not found to be significantly altered in our qPCR-based validation, in contrast to our sequencing data. Accordingly, only the miRNAs with consistently altered expression levels were selected for further study.

### Correlation between relative expression levels of miRNAs and germ cell apoptosis

**Figure 7:**
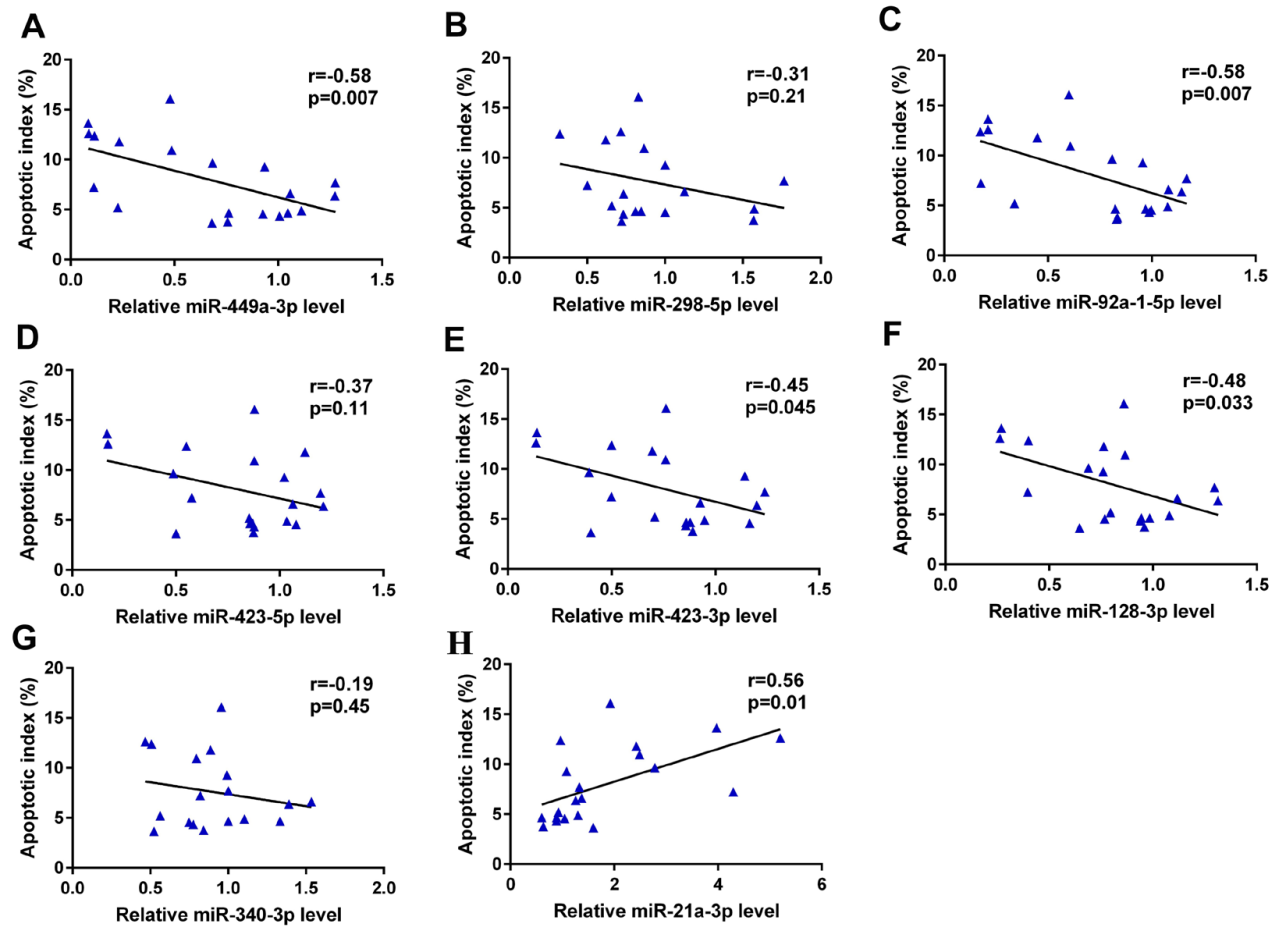
Correlation between relative expression levels of miRNAs and germ cell apoptosis The relative levels of miR-449a-3p **(A)**, miR-92a-1-5p **(C)**, miR-423-3p **(E)** and miR-128-3p **(F)** correlated significantly and negatively with the germ cell AI (r = -0.58, -0.58, -0.45, and -0.48, respectively; *p* = 0.007, 0.007, 0.045, and 0.033, respectively); the relative level of miR-21a-3p **(H)** correlated significantly and positively with the AI (r = 0.56, *p* = 0.01). The relative levels of miR-298-5p **(B)**, miR-423-5p **(D)** and miR-340-3p **(G)** were not significantly correlated with germ cell AI. AI, apoptotic index.

We next performed a Spearman correlation analysis to analyze the relationship between relative miRNA levels and the germ cell AI. We found that the relative levels of miR-449a-3p, miR-92a-1-5p, miR-423-3p and miR-128-3p correlated significantly and negatively with the germ cell AI (r = -0.58, -0.58, -0.45, and -0.48, respectively; *p* = 0.007, 0.007, 0.045, and 0.033, respectively). Furthermore, the relative level of miR-21a-3p correlated significantly and positively with the AI (r = 0.56, *p* = 0.01). In summary, these 5 miRNAs might participate in the process of scrotal hyperthermia-induced germ cell apoptosis (Figure 7).

## DISCUSSION

To our knowledge, this is the first study to identify differentially expressed small RNAs in heat-treated testes. Through our attempt to delineate the early testicular miRNA response to heat treatment and elucidate the role of miRNAs in the heat-induced suppression of spermatogenesis, we identified germ cell apoptosis as the main event in heat-induced spermatogenic impairment. However, the molecular mechanism underlying early-stage germ cell apoptosis remains unclear.

Consistent with a study published by Amiya et al. [18], we did not observe obvious histologic damage to the testes at 6 hours after scrotal hyperthermia, despite the presence of many more apoptotic germ cells. Accordingly, we selected this time point for the identification of differentially expressed miRNAs. Our high-throughput sequencing results, obtained in triplicate, identified 11 miRNAs as differentially expressed after scrotal hyperthermia (novel miRNAs were not involved). As miRNAs exert their functions by regulating target mRNAs, the targets must be identified before the function can be understood. Our miRNA enrichment analysis revealed that the target genes of the differentially expressed miRNAs in our study are involved in various processes, including the ribosome, HIF-1 signaling pathway (i.e., hypoxia), endocytosis, and MAPK signaling pathway.

Regarding the identified pathways, the ribosome, which acts as a protein production factory, is sensitive to acute stress. Notably, the functional status of the ribosome is crucial for cellular homeostasis and genetic integrity [21]. Hypoxia occurs when the blood oxygen pressure decreases below the level required for normal cellular function in a particular tissue. Studies have revealed that increased metabolism in the testis after heat stress may not be compensated by a sufficient increase in blood flow [22], and increased HIF-1ɑ mRNA expression has been detected in mouse testes subjected to transient scrotal hyperthermia [23, 24], as well as in sperm collected from men exposed to a sauna [6]. A previous cross-species study by Vera et al. reported the involvement of the MAPK pathway during apoptotic signaling within male germ cells subjected to hormone deprivation [25], and Jia et al. demonstrated a critical role for MAPK14 in heat stress-induced germ cell apoptosis [26]. In light of these earlier findings, our results suggest that the differentially expressed miRNAs identified in this study may regulate target genes involved in multiple pathways related to heat stress-induced spermatogenic impairment.

A total of 8 miRNAs were validated to be consistent with the differentially expressed miRNAs identified by the sequencing, whereas another 3 miRNAs were not. This inconsistency may be due to the different methodology. Our observation that miR-298-5p was downregulated after scrotal hyperthermia was not previously reported in studies of testicular miRNA expression. However, Barbagallo et al. reported that the downregulation of miR-296-3p and miR-298-5p, which consequently led to the upregulation of their respective targets IGF1Rβ and TNFα, was a major determinant of the resistance of mammalian pancreatic α cells to cytokine-induced apoptosis [27]. Nevertheless, we did not observe the differential expression of miR-296-3p after scrotal hyperthermia. Furthermore, our observation of a non-significant association between the relative miR-298-5p level and the germ cell AI in the correlation analysis suggests that the downregulation of miR-298-5p alone may not be sufficient to induce germ cell apoptosis.

Furthermore, our report is the first to identify the downregulation of miR-423-5p in the testis in response to scrotal hyperthermia. Previously, Wan et al. reported that miR-423-5p knockdown correlated with reduced proliferation and increased mitochondria-dependent apoptosis among glioma stem cells [28]. Other studies found that miR-423-5p regulates gastric cancer cell proliferation and invasion [29] and promotes hepatocarcinoma cell autophagy [30]. Altogether, our and previous findings have led us to hypothesize that miR-423-5p downregulation may contribute to heat stress-induced spermatogenic impairment, as increased germ cell apoptosis and suppressed germ cell proliferation were identified as the main mechanisms leading to heat-stress induced testicular damage [31, 32].

Similarly, our report is the first to describe the significant downregulation of miR-128-3p expression in the testis after scrotal hyperthermia. Previously, Mao et al. reported that miR-128-3p directly targets the *MAPK14* 3’UTR and downregulates MAPK14 protein expression; furthermore, an increased miR-128-3plevel was found to contribute to neuronal survival in an ischemia-induced brain injury [33]. MAPK14, a pro-apoptotic protein, was shown to be upregulated in germ cells, where it activates the mitochondrial-dependent apoptotic pathway by provoking BCL2 phosphorylation or altering the BAX/BCL2 expression ratio in testes subjected to heat stress or hormonal deprivation [26]. Collectively, these findings suggest a critical role for miR-128-3p in maintaining normal spermatogenesis. Furthermore, the heat-induced downregulation of miR-128-3p may promote germ cell apoptosis by up-regulating MAPK14, which was supported by finding of a significantly negative association between relative miR-128-3p level and the germ cell AI in a correlation analysis (r = -0.48, *p* = 0.033).

Finally, we validated the differential expression levels of miR-449a-3p, miR-92a-1-5p, miR-423-3p, miR-340-3p, and miR-21a-3p, which were in agreement with our sequencing results. Nevertheless, no previous study has reported the expression of these miRNAs in the testis or associations with stress-related functions. We further identified significant associations of miR-449a-3p, miR-92a-1-5p, miR-423-3p, and miR-21a-3p with the germ cell AI, suggesting that these miRNAs may directly or indirectly regulate apoptosis-related pathways.

In summary, ours is the first study to investigate the roles of miRNAs in heat stress-induced spermatogenic impairment. Using high-throughput sequencing, we identified 11 miRNAs exhibiting differential expression during an early stage of testicular damage. Notably, the testicular expression of most of these miRNAs had not previously been reported. Moreover, we demonstrated the involvement of these identified miRNAs in various pathways (e.g., ribosome, HIF-1, MAPK) that may be critical to heat stress-induced testicular damage. Finally, some of the identified miRNAs (e.g., miR-449a-3p, miR-92a-1-5p, miR-423-3p, and miR-128-3p) correlated closely with germ cell apoptosis. Further study is needed to clarify the detailed mechanism by which these miRNAs regulate their targets and thus affect spermatogenesis.

## MATERIALS AND METHODS

### Animals and treatment

Male adult ICR mice (age: 7–8 weeks) were purchased from the Hubei Provincial Center for Disease Control and Prevention, and were housed under standard conditions with free access to water and food. The animal protocol was approved by the Institutional Committee on Animal Care and Use.

Thirty mice were randomly divided into control and heat treatment groups. Each mouse from the latter group were subjected to a single heat stress of 43°C for 30 min, as described in another study [34]. Briefly, the lower part of the body including hind legs, tail and scrotum was submerged in a thermostatically controlled water bath at 43°C for 30 min, under anesthesia (intraperitoneal injection of 1% pentobarbital sodium at 50 mg/kg body weight). After heat treatment, the animals were dried, examined for any injury to the testes, and returned to their cages. No injuries to the mice’s scrotal skin were found during this process. No intervention was conducted on mice in the control group.

All animals were sacrificed 6 hours after treatment. The left testes were immediately stored in liquid nitrogen and later used for small RNA sequencing. The right testes from 10 mice per group were fixed in Bouin’s solution for apoptosis assessment.

### Assessment of germ cell apoptosis

Sections from paraffin-embedded testes samples were subjected to germ cell apoptosis analysis, using a terminal deoxynucleotidyl transferase (TdT)-mediated deoxyuridine triphosphate (dUTP) nick-end labeling (TUNEL) assay with an Apop Tag-peroxidase kit (EMD Millipore international Inc.), as described in our previously published study [7]. Briefly, the sections were dewaxed and hydrated, treated with H_2_O_2_ to quench endogenous peroxidase activity. After washing with PBS, labeling was performed by incubation for 1 h at 37°C with 50 μl of a labeling solution containing deoxyuridine triphosphate (dUTP) and terminal deoxynucleotidyl transferase (TdT) and 50 μl of streptavidin–horseradish peroxidase solution, and the samples were counterstained with hematoxylin, dehydrated, and mounted. This assay included a negative control in which the TdT enzyme was omitted from the reaction system, and a positive control treated with DNase before the TUNEL assay. Subsequently, the apoptotic index (AI) of each sample was calculated as described in another study [26]. Briefly, the non-apoptotic Sertoli cell nuclei with distinct nucleoli and the apoptotic germ cell population were enumerated in all seminiferous tubules using an Olympus microscope (Olympus, Tokyo, Japan) at 200× magnification. The AI was expressed as the number of apoptotic germ cells per 100 Sertoli cells [26, 35]. Ten animals were used per group.

### RNA extraction and qualification

TRIzol reagent (Invitrogen/Life Technologies, Carlsbad, CA, USA) was used to extract total RNA from the testes according to the manufacturer’s protocol. RNA purity was assessed using a Kaiao K5500 spectrophotometer (Kaiao, Beijing, China). RNA integrity and concentration were assessed using an Agilent 2100 RNA Nano 6000 Assay Kit (Agilent Technologies, Foster City, CA, USA). We used a sample mix strategy to reduce individual differences among samples. In brief, equal amounts of total RNA from every 5 mice per group were pooled to yield a total of 3 pooled samples per group (n = 3) for small RNA sequencing.

### Library preparation and sequencing

Small RNA libraries were prepared using the TruSeq Small RNA Sample Preparation Kit (Illumina Inc., San Diego, CA, USA) according to the manufacturer’s protocol. One microgram of RNA per sample was subjected to RNA 3′ adapter ligation, RNA 5′ adapter ligation, cDNA synthesis, PCR amplification with unique barcode sequences for each sample, and gel size-selection (15–35 nt) for the small RNA library. The yields of sequencing libraries were assessed using an Agilent 2100 Bioanalyzer (Agilent Technologies). Multiplexed libraries were sequenced on a HiSeq2500 (Illumina) next-generation sequencing platform.

### Bioinformatics analysis of small RNA-seq data

We performed several filtering steps after obtaining and demultiplexing the raw reads. Reads that met the following filtering criteria were removed: 1) no 3’ adapter, 2) no insert fragment, 3) excessively long poly A/T sequence, 4) length outside of the specified range, or 5) low quality. A low-quality read was defined as a read in which >15% of the read bases had a Phred Quality value (the error rate of each base sequencing) of ≤19. We also removed reads containing > 5% of N bases among the total. After filtering, we obtained clean reads for the quality and data quantity, including Q20 (error rate of base sequencing <0.1) and Q30 (error rate of base sequencing <0.01) statistics, data quantity statistics, and base content statistics. The reference genome library was constructed using Bowtie 1.0.1 software, and clean data were mapped to the reference genome.

### miRNA identification

Briefly, known miRNA identification aims to achieve overlap (100%) between the genome locations of mapping reads and of reference miRNAs. Genome annotation data available in miRBase (Release 21) was subjected to an overlap analysis using Bedtools software. Otherwise, reference miRNA must be mapped to the reference genome to obtain location information before an overlap analysis can be performed. After excluding reads that mapped to known miRNA/non-coding RNA/repeat regions/mRNA regions, the remaining reads were used to predict novel miRNAs using miRDeep2 software. The stacking of reads that formed hairpin structures was key to identification and evaluations of potential importance and stability.

### Differential miRNA expression analysis

For every sample, the miRNA count and reads per million total reads (RPM) were used to normalize the expression levels of specific miRNAs for direct use in inter-library comparisons. DESeq software (v1.16.0) and a model based on the negative binomial distribution were used for the differential gene expression analysis of control and heat-treated samples. A p-value was assigned to each miRNA, and the Benjamini and Hochberg approach was used to adjust these values and control the false discovery rate. MiRNAs with *p* values <0.05 and |log2 (fold-change)| values ≥1 were considered differentially expressed.

### miRNA target prediction and GO and KEGG analyses

miRanda software (3.3a) was used in this study to predict the targets of known or novel miRNAs. The hypergeometric test, in which the *p*-value is calculated and adjusted to yield a *q*-value, was used to assess the GO (Gene Ontology) enrichment of miRNA target genes against the background of the whole genome. The GO analysis provides three basic types of information: Biological Process (BP), Cell Component (CC), and Molecular Function (MF). GO terms with *q* values <0.05 were considered significantly enriched.

KEGG (Kyoto Encyclopedia of Genes and Genomes, http://www.kegg.jp/) is a database of manually drawn pathway maps representing currently known molecular interactions and reaction networks. To determine the KEGG enrichment of target genes, we implemented the hypergeometric test, in which *p*-values were adjusted to *q*-values using multiple comparisons. KEGG terms with *q* values <0.05 were considered significantly enriched.

### Validation of the miRNA sequencing results

Eleven miRNAs were found to exhibit significant differential expression, and all were selected for qRT-PCR-based validation, as described in another study [36]. Briefly, total RNAs extracted from testes were subjected to reverse transcription using oligo (dT). Specific primers for RT-qPCR were designed using Primer 5.0 software, and the sequences are listed in Supplementary Table 1. Real-time qPCR analyses were performed in triplicate using SYBR Green (Takara, Shiga, Japan) on a Light Cycler® 96 System (Roche, Basel, Switzerland). U6 snRNA was selected as an internal control for miRNA expression. Ten animals were used per group (control and heat treatment). The cycle threshold (Ct) value obtained for each target gene was normalized to the Ct value for U6 using the comparative Ct method [37].

### Statistical analysis

The AIs and relative miRNA expression levels are expressed as means ± standard deviations. The Mann–Whitney U-test was used to compare the AIs and relative miRNA expression levels before and after hyperthermia. A Spearman correlation analysis was performed to evaluate the correlations of miRNA expression levels with germ cell apoptosis. The statistical analysis was performed using SPSS 17.0 software (SPSS Inc., Chicago, IL, USA). A *p* level of <0.05 was considered to indicate statistical significance.

## SUPPLEMENTARY MATERIALS FIGURE AND TABLE



## Abbreviations

MiRNA: micro RNA
GO: gene ontology
KEGG: Kyoto Encyclopedia of Genes and Genomes
AI: apoptotic index
HIF-1: hypoxia-induced factor 1
MAPK: mitogen-activated protein kinase
