# Supplementary material for: Next-generation sequencing-based microRNA profiling of mice testis subjected to transient heat stress
Source: Oncotarget. 2017 Dec 4;8(67):111672–82. doi: 10.18632/oncotarget.22900 (PMC5762351; doi:10.18632/oncotarget.22900)
Supplement: Supplementary file 1 [file oncotarget-08-111672-s001.pdf]

Next-generation sequencing-based microRNA profiling of mice testis subjected to transient heat stress

SUPPLEMENTARY MATERIALS

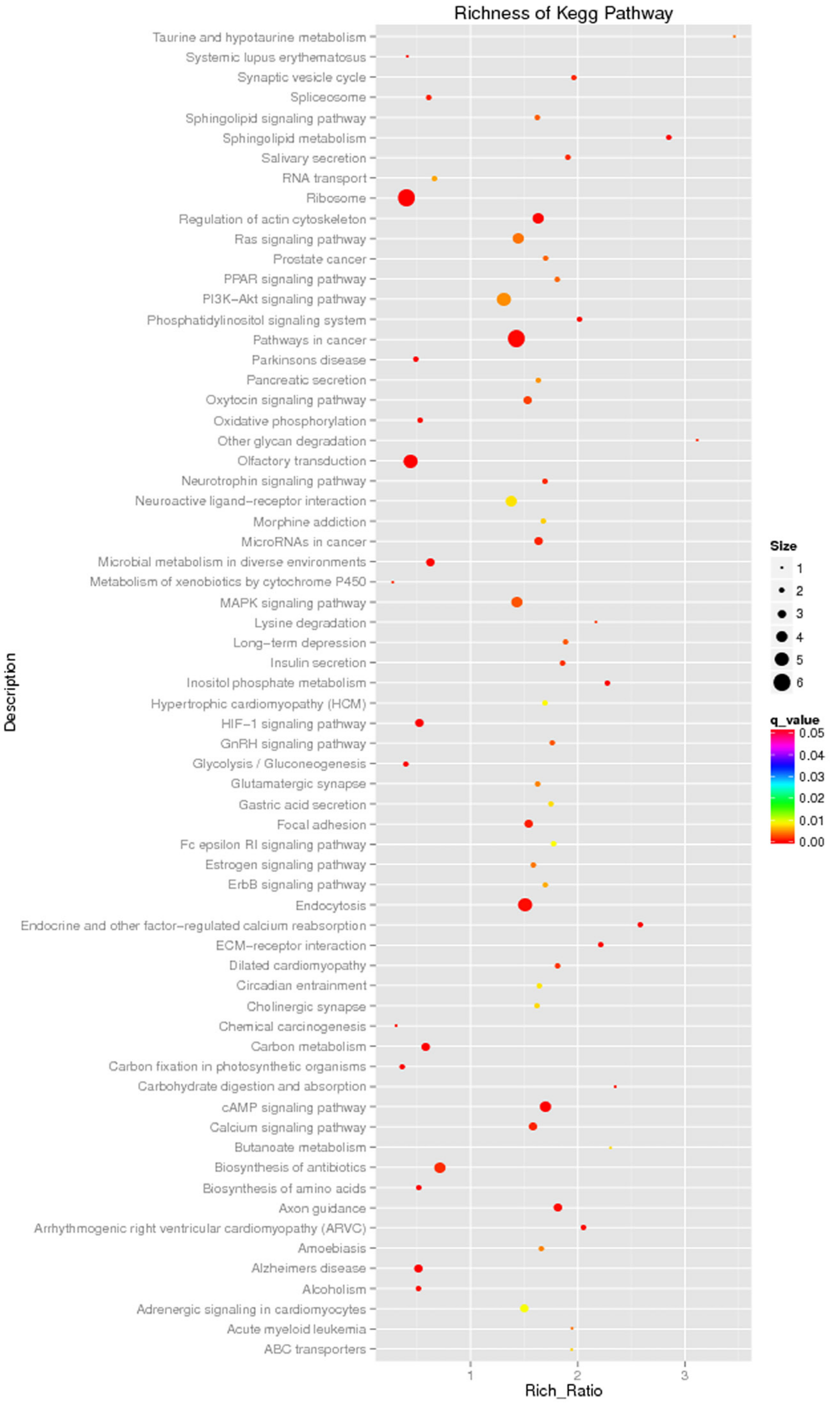

Supplementary Figure 1: Kyoto Encyclopedia of Genes and Genomes (KEGG) analysis to enrich the potential pathways of the target genes. The y-axis represents the name of the pathway, and the x-axis represents the Rich factor. Dot size represents the number of different genes and the color indicates the q-value.

Supplementary Table 1: Primer sequences used for qRT-PCR

Gene name	RT primer(5' to 3')	Forward Primer(5' to 3')	Reversed Primer(5' to 3')
miR-449a-3p	CCTGTTGTCTCCAGCCACAAAAGAGCACA ATATTTCAGGAGACAACAGGGAGAGCA	CGGGCCAGCTAACATGCGAC	CAGCCACAAAAGAGCACAAT
miR-298-5p	CCTGTTGTCTCCAGCCACAAAAGAGCACA ATATTTCAGGAGACAACAGGGGGAAGA	GGGCGGCAGAGGAGGGCTGT	CAGCCACAAAAGAGCACAAT
miR-92a-1-5p	CCTGTTGTCTCCAGCCACAAAAGAGCACA ATATTTCAGGAGACAACAGGAGCATTG	CGCCGAGGTTGGGATTTGTCG	CAGCCACAAAAGAGCACAAT
miR-423-5p	CCTGTTGTCTCCAGCCACAAAAGAGCACA ATATTTCAGGAGACAACAGGAAAGTCT	CGCCGTGAGGGGCAGAGAGCG	CAGCCACAAAAGAGCACAAT
miR-423-3p	CCTGTTGTCTCCAGCCACAAAAGAGCACA ATATTTCAGGAGACAACAGGACTGAGG	GCGGCAGCTCGGTCTGAGGCC	CAGCCACAAAAGAGCACAAT
miR-128-3p	CCTGTTGTCTCCAGCCACAAAAGAGCACA ATATTTCAGGAGACAACAGGAAAGAGA	CGGGCTCACAGTGAACCGG	CAGCCACAAAAGAGCACAAT
miR-340-3p	CCTGTTGTCTCCAGCCACAAAAGAGCACA ATATTTCAGGAGACAACAGGGCTATAA	GCGGCTCCGTCTCAGTTACT	CAGCCACAAAAGAGCACAAT
miR-98-5p	CCTGTTGTCTCCAGCCACAAAAGAGCACA ATATTTCAGGAGACAACAGGAACAATA	CGGGCTGAGGTAGTAAGTTG	CAGCCACAAAAGAGCACAAT
miR-3968	CCTGTTGTCTCCAGCCACAAAAGAGCACA ATATTTCAGGAGACAACAGGTGGTGTC	CGCCGCGAATCCCACTCCA	CAGCCACAAAAGAGCACAAT
miR-21a-3p	CCTGTTGTCTCCAGCCACAAAAGAGCACA ATATTTCAGGAGACAACAGGGACAGCC	CGCCGCAACAGCAGTCGATG	CAGCCACAAAAGAGCACAAT
miR-201-3p	CCTGTTGTCTCCAGCCACAAAAGAGCACA ATATTTCAGGAGACAACAGGCCTACAC	CGGGCTGAACAGTGCCTTTCT	CAGCCACAAAAGAGCACAAT
U6	ACGCTTCACGAATTTGCGTGTC	CTCGCTTCGGCAGCACATATACT	ACGCTTCACGAATTTGCGTGTC
